# Health Tract, No. 247

**Published:** 1866-06

**Authors:** 


### HEALTH TRACT, No. 247.

### FILTH AND PURITY.

If, on some cloud chariot in rosy June, the reader could be transported across continents and seas, and alight amidst one of the villages of the Ferroe Islands, off Scotland, he would find a condition of noisomeness and filth around the dwellings, which could not be equalled in any village community on the face of the globe; yet, in all the wide world, there is not a people that enjoys such an exemption from sickness and death; only twelve out of a thousand die in a year; but twenty-four out of every thousand of the population, die in the United States, annually.

Not long ago, a malignant disease appeared in a farmer's family; every circumstance compelled the intelligent physician to believe that it had a local origen; but trees, and lawn and garden, with whitened fences, showed that there was industry and thrift, and elevation in that old homestead; but upon a vigilant inspection, a depression was found not far from the kitchen door, into which every basin of water, whether from washing the hands, the dishes or milk pans, found its way, after it was dashed out from the kitchen door; the soil was saturated with it, but no odor was observed to arise from it.

When the Paris authorities ordered a grave yard to be dug up, many bodies were found to have been converted into what was called Adipocre. The stench was such that some of the workmen fainted, and but few could keep their places more than half an hour at a time, when they had to rush into the pure air; yet not a single case of disease occurred during the several weeks the operations were continued.

When we lived in New Orleans many years ago, we knew, if any epidemic was prevailing, whether cholera, or yellow or congestive fevers, and the atmosphere of sundown and early morning was peculiarly balmy, and seemed as pure and sweet as angel zephyrs, that the disease would become more malignant for several days afterwards; proving the before known fact, that the cause of fever in the air—marsh miasm—was not perceptible to the senses. The beautiful consistency of these apparently most contradictory facts, shows at once the goodness and wisdom of our common Father in Heaven; the very sight of filth and accumulations of house and kitchen offal is demoralizing, hence such an offensive odor is connected with it, as to compel a greater or less attention to its removal or abatement. The destruction of all vegetable products is necessary, as a fertilizer; the gas of these, marsh miasm, is free from smell, and man's higher powers of reason are brought into requisition to search out and counteract these disease engendering influences. The Ferroe Islanders live by fishing; from May to November their villages are entirely deserted, and they live upon the sea, inhaling day and night its pure and luscious air. In winter, averything is frozen stiff and remains so, hence there are no odors and no decay. As to the infected farm house, the prevailing wind was from the filth-saturated depression towards the house, and this air was breathed day and night. Heat rarifies all noxious gases and odors, and sends them to the clouds; these are most pernicious at sunrise and sunset, hence building fires in the family sitting room at those hours, will, other things being equal, exempt families from epidemics, chills and fevers and perhaps even cholera itself.

